# Pennsylvania State Veterinary Medical Association

**Published:** 1895-10

**Authors:** F. S. Allen

**Affiliations:** Acting Secretary


Secretary.

### PENNSYLVANIA STATE VETERINARY MEDICAL ASSOCIATION.

The semi-annual meeting was held in the Mountain House, at Cresson Springs, September 3, 1895. The meeting was called to order at 10.30 A.M. by the President, Dr. Leonard Pearson. In the absence of the Recording Secretary, Dr. W. G. Benner, a motion was made and carried that Dr. F. S. Allen, the Corresponding Secretary, act also as Recording Secretary. At roll-call the following members were found to be present: Dr. Jacob Helmer, Dr. John R. Hart, Dr. Leonard Pearson, Dr. James B. Rayner, Dr. Thomas B. Rayner, Dr. Otto Noack, Dr. J. H. Timberman, and Dr. F. S. Allen. The minutes were then read and approved, with the correction that a list of all members dropped at the last meeting, March, 1895, for non-payment of dues, be added to the minutes. There were three new members proposed for admission to the Association: Dr. George W. Bell, Dr. George B. Jobson, and Dr. Daniel C. Gearhart. In the absence of Dr. J. C. McNeil, of the Board of Censors, the President appointed Dr. Jacob Helmer to act with the Board. The meeting then adjourned for fifteen minutes while the Board considered the names of the new applicants for membership.

Meeting called to order. Dr. W. H. Hoskins, Chairman of the Board of Censors, reported favorably the names of Dr. George W. Bell and Dr. Daniel C. Gearhart. The name of Dr. George B. Jobson was laid over to the next meeting, with the request that Dr. Jobson be notified and requested to appear at that time before the Board. Motion made and seconded that the report be accepted; carried. Motion was then made that the Secretary cast a ballot in favor of the two new members; carried; and Drs. George W. Bell and Daniel C. Gearhart became members. The report of the Corresponding Secretary was then made as follows:

"At the last annual meeting of this Association there were one hundred and twenty-six members enrolled, and to this number were added six new members: Dr. W. T. S. Werntz, Dr. H. J. McClellan, Dr. John F. Vandegrift, Dr. Edward L. Kellner, Dr. Charles Bland, and Dr. E. E. Terry, making in all one hundred and twenty-nine active and three honorary members. At the close of the last meeting it was ordered by this Association that fourteen members be suspended for the non-payment of dues, and that each member suspended be notified of the same, and, if possible, induced to remit the amount of his indebtedness. Your Secretary has accordingly sent out bills twice during the past six months to the suspended members, and each time enclosed a letter urging each member to settle his account and thus continue as member of this Association, also assuring him that the Association regretted exceedingly the necessity of being obliged to take this step, but that it was but just and right that all members should join in defraying the necessary expenses of the Association; that the Association had been under a great expense during the past year in its efforts to secure the passage of the two bills that have recently become laws; and, further, that no Association could continue to do good work without the necessary funds. To these appeals there has, as yet, been no response. Thus the membership has been reduced to one hundred and fifteen active members. Still there are many who should be suspended. Some have received their certificates and decline to respond to all notices sent out. There are also five certificates ready for delivery. Although not less than four notices have been sent out to each member during the past six or eight months, I have been unable to obtain a response. A certificate has been ordered for Dr. S. D. Larzelere, who became a member in March, 1893, and is in good standing, but has never received a certificate. Dr. Thompson has been notified that if he would remit the amount due his certificate would be forwarded to his address. Dr. Samuel B. Willard has also been notified to send in his application and remit the amount due and his certificate would be forwarded. Neither gentleman has responded, although two notices have been sent out. Dr. George B. Jobson was duly notified to appear at this meeting, to which he promptly responded, giving good and sufficient reasons for not being able to attend this meeting, and furnishing a paper of his experiences with abortion. All new members have been duly notified that their certificates would be ready at this meeting on payment of initiation fees, etc.

"To all members of this Association two notices of this meeting have been sent. Bills have also been rendered to all the members, and to those in arrears for more than a year letters have been enclosed. Some few have responded."

A motion was seconded that the report be accepted; carried. Dr. John R. Hart and Dr. Allen were requested to examine the accounts of the Association. The Treasurer then made a report or statement of the financial condition of the Association, showing a balance in the treasury, with a large amount outstanding against the members. A discussion then ensued, in which nearly all member present participated, as to the propriety of dropping all the delinquent members. As a result of this discussion Dr. Pearson offered the following amendment to Art. V., Sec. 2 of by-laws: "That the Recording Secretary shall keep an accurate record of the attendance of the meetings—at all regular meetings—and after three successive absences the members shall be sent a copy of Article V., Section 2, and informed that the above section will be enforced." Motion seconded and carried. A motion was made by Dr. John R. Hart that all members in arrears for one or more years shall be notified that the claim against him will be placed in the hands of a collection agency, and if not paid within sixty days the Corresponding Secretary shall see that suit is entered and carried out. Motion seconded and carried. Dr. Thomas B. Rayner made a motion that a committee be appointed to draft resolutions and forward the same to Mr. William H. Woodring, of Northampton County, to show our appreciation of his efficient and untiring services during the past winter in behalf of this Association by assisting the Legislative Committee in their efforts to secure the passage of the two bills presented to the last Legislature by this Association, and which are now laws of the State. The motion was seconded and carried. The President appointed Dr. T. B. Rayner, Dr. John R. Hart, and Dr. W. Horace Hoskins to act on this committee. Dr. W. H. Hoskins then made a motion that a delegation be appointed to the United States Veterinary Medical Association, to be held in Des Moines, Iowa, September 10th, 11th, and 12th. Motion carried. The following were appointed by the President: Dr. T. B. Rayner, Dr. Jacob Helmer, Dr. John R. Hart, Dr. J. B. Rayner, and Dr. J. C. Foelker.

Dr. W. H. Hoskins then made his report as Chairman of the Committee on Legislation:

"While I strongly importuned our President to place some other member at the head of the Legislative Committee for the present year, it was his wish that I should remain in the direction of this work until the legislation under consideration the past two years should become fixed laws in our Commonwealth, and the promise that there was little left to be done led to my acceptance of this post of duty.

"When we met in March last in Philadelphia the two acts which we had pressed so hard for consideration were in the last steps of their accomplishment, and, as you all know, they were finally adopted by both branches of the Legislature, and subsequently received the Governor's approval, and are now fixed statutes of the State, but still in a very unsatisfactory and uncertain condition. The act establishing a State Live Stock Sanitary Board, which was to have gone into effect on June 1st last, still slumbers, and we should endeavor to awaken by some means to-day the officials who are charged with its execution. But two of those who are to be members of this Board have received their appointment, and the one important position to our people generally, to us specially, still remains unnamed or appointed. While we had every reason to believe that the duties of this gravely important position would fall on the shoulders of our worthy President, and of whom it may be said with one accord that to none would this position come in whom the veterinary profession the length and breadth of the State would feel more confidence, and believe that through him the highest mission of this legislation for our people would be best accomplished, we have with one accord been sorely pained to realize that the exigencies of politics—the baneful perpetuation of the infamous spoils-system—has robbed our State of his valuable services for the time, and our people of the defense and protection this Board promised to assure them and the profession in the Keystone State of the possession of a wise law, conservative plan, and valued measure alongside our colleagues of other States in dealing with these portentous questions of so much import to the health and happiness of our people. We still hope and believe that our Governor will make this appointment, and thus bring to his administration in this specific direction the valued services of one thoroughly equipped for this place in my estimation, and through whom there will inure to his administration, the people, and our profession the greatest good to the greatest number, the true and highest attainment of all legislation for the people, of the people, and by the people.

"The second act, establishing a State Board of Veterinary Medical Examiners, after some delay and slight changes, passed both Houses, and, receiving the Governor's signature, became a law. Its provisions called for its enforcement on September 2d, when was designated the time for the convening and organization of the Board. The appointments of this Board have also been denied us, for the reasons named applying to the office of State Veterinarian and the Live Stock Sanitary Board. May the lessons that these experiences teach us, that the day of offensive partisanship has reached such a climax that for the good interests of our people it must be wiped out as a blot upon our country and an insult to the intelligence of our people. We are a part of the people against whom these charges rest, and we are factors of influence and power in the better solution of these questions; let us then never lose the opportunity of so casting our ballot that we can be charged with wearing a party yoke, or of yielding our virtues and manliest traits of independence, by conceding our party slavery, when we cast a ballot for a bad man, who by his machinations, machine nominations, or combine allotments may have obtained a place on the ticket that stands for the party which best represents the principles we uphold. These appointments should have been made many months ago, that the work charged to the execution of this law should have been complied with as intended by its provisions and our lawmakers.

"Our people are entitled to all the benefits of legislative enactments, and a long-suffering public arrived at exact conclusions when her unqualified support was given to these measures through the campaign of education instituted by this organization and completed through the earnest, conscientious work of its members, directed and supported at all times and at all hours by the untiring energy and devotion of our President. Therefore the denial of these benefits and security to the people of our State comes to them as a condition of affairs for which the weakest excuse that intelligent officials may offer would be those of a partisan, factional, political character.

"During the past six months several violations of the State law have been brought to our notice, and vigorous action upon the part of several of our veterinarians led to summary action and relief from these parasites of the profession.

"In Allegheny County two travelling peripatetic veterinary dentists squatted themselves for a time to gull an innocent, confiding public by their arrogance and charlatanism, when, through the efforts of Messrs. Waugh and Rechtenwald, under the direction of your committee, warrants were sworn out for their arrest. Through some weakness of the machinery for the execution of our laws these fellows learned of the action against them and suddenly left for new fields and pastures green.

"In Washington County action was commenced against another veterinarian for practice without registration, and was settled by the imposition of a fine and the removal of the offender to other parts.

"A third case coming under our notice was the location in Reading of a veterinarian named McNeil, claiming to be a graduate of Harvard Veterinary Department and the only veterinarian in Reading honored with membership in the United States Veterinary Medical Association. He had gone so far as to make a registry of those facts on the prothonotary record. Dr. Noack kindly reported the case with a newspaper comment of his talents, which was a tissue of falsehood and misstatements. Investigation was promptly made, and the courtesy of Prof. Osgood quickly armed us with all the necessary evidence, upon receipt of which I wrote him a very firm and emphatic letter as to the false statements he had entered upon the county register, and that if the same was not corrected within forty-eight hours from the receipt of my letter prosecution would be commenced, whereat he disappeared promptly and failed to leave his new postoffice address.

"Such is, in brief, a few points of action relating to legislation during the past six months; and with such vigorous action in the enforcement of our new laws, we soon would find that no State in our country was more highly blessed in the attainment of wise and just laws, all tending to a higher and better condition of affairs, a more exalted standard for our profession, a higher recognition of the importance to the well-being of every community, and a more generously conceded support and sustenance of our profession, than Pennsylvania."

Motion made and accepted that the report be accepted as read. Seconded and carried.

Dr. M. E. Conard, Chairman of the Committee on Sanitary Science and Police, being unable to attend, placed his report in the hands of the President:

"We, the members of the Committee on Sanitary Science and Police, beg to make the following report: Since our last annual meeting (March 3, 1895) there has been an unusually small amount of disease of a contagious character reported to me, and I believe likewise to my fellow-committeemen. In the early part of this year, as you all know, there were legislative steps taken toward the inspection of live stock, with compensation for those animals destroyed on account of the presence of contagious diseases. The appointment of the officials necessary to the enforcement of the very just and valuable law has been deferred beyond the time when it was generally looked for by the political contest just now passed. These conditions I believe have had the tendency, at least in many localities, to suppress the information necessary to a full report of our subject. If this be the case, as I believe it is, we should be enabled to give a much fuller report at our next annual meeting in March, 1896. But by the kindness of Dr. Bridge and others of the committee I am enabled to make the following report.

"During the past six months there have been forty-one outbreaks of glanders reported, in which 64 horses and mules were affected, 59 of which have been destroyed; 51 have been paid for by the State at an average of $16 per head. Several herds of cattle affected with tuberculosis have been inspected and tested with tuberculin, the diseased members destroyed and paid for by the State. This, however, has only been done where herds were badly affected and their products were sold in the large cities.

"There have been three outbreaks of anthrax in herds numbering 220 head of cattle—95 have died.

"One new outbreak of foot-rot in a flock of Dorset sheep has been reported; the affected ones have all been killed and the others thoroughly disinfected, and it is believed to be stopped. The flock of Dorset sheep reported by me at our last meeting as affected with foot-rot have been almost all killed for mutton, leaving only those that are surely free from the disease.

"Six outbreaks of Texas fever, with a fatality of 28 cattle out of 70 head exposed. All cattle from localities known or believed to be infected with the Texas tick should be subjected to a thorough disinfection before being allowed to come in contact with suspected herds; perhaps a strong solution of creolin might do, but it should be something that would surely kill all ticks on them.

"In addition we have all had our usual outbreaks of influenza, distemper, strangles, etc., incident to the season in which so many horses are shipped from the West.

"I hope this very meagre report may at least answer for an apology in my absence."

Dr. John Adams, the Chairman of the Committee on Intelligence and Education, was unable also to be present owing to sickness in his family. No report was furnished.

A lengthy discussion then ensued, in which nearly all present took part, in regard to the long-deferred appointment of the State Veterinarian.

Dr. W. H. Hoskins then suggested that Dr. John Hart be appointed or asked to wait upon the Governor, and to solicit an interview and look after the appointment of the State Veterinarian. Discussion then followed.

Motion made to adjourn until 2 o'clock. Carried.

*Afternoon Session.*—Dr. W. H. Hoskins made extended remarks as to the health of Dr. S. E. Weber, of Lancaster, Pa., stating the critical as well as serious condition in which he is in, as witnessed by him, and asked that a fund be raised by the members of this Association to assist him and to show our high appreciation of this most honored and esteemed fellow-member. Dr. John Hart then started the collection, to which all the members present responded by placing the money in the hands of Dr. W. H. Hoskins.

The next in order was the reading of papers. In the absence of Dr. James Waugh, his paper on "Paracentesis Abdominalis"[1] was read by the Secretary. Dr. Otto Noack then read a very interesting paper on "Trichinosis."[2] Dr. Jacob Helmer also read a lengthy and well-prepared paper on "Physical Diagnosis."[3] In the absence of Dr. C. Bland his paper on "Tips and Shoeing"[4] was also read by the Secretary. Dr. S. J. I. Harger forwarded a paper on "Serum Therapeutics,"[5] which was listened to with marked attention.

The meeting then adjourned, and the members left for Philadelphia on the 8.12 P.M. train.

F. S. ALLEN,
Acting Secretary.

